# VALENCIA: a nearest centroid classification method for vaginal microbial communities based on composition

**DOI:** 10.1186/s40168-020-00934-6

**Published:** 2020-11-23

**Authors:** Michael T. France, Bing Ma, Pawel Gajer, Sarah Brown, Michael S. Humphrys, Johanna B. Holm, L. Elaine Waetjen, Rebecca M. Brotman, Jacques Ravel

**Affiliations:** 1 Institute for Genome Sciences, University of Maryland School of Medicine, Baltimore, MD USA; 2 Department of Microbiology and Immunology, University of Maryland School of Medicine, Baltimore, USA; 3 Department of Epidemiology and Public Health, University of Maryland School of Medicine, Baltimore, USA; 4 Department of Obstetrics and Gynecology, University of California Davis School of Medicine, Sacramento, USA

## Abstract

**Background:**

Taxonomic profiles of vaginal microbial communities can be sorted into a discrete number of categories termed community state types (CSTs). This approach is advantageous because collapsing a hyper-dimensional taxonomic profile into a single categorical variable enables efforts such as data exploration, epidemiological studies, and statistical modeling. Vaginal communities are typically assigned to CSTs based on the results of hierarchical clustering of the pairwise distances between samples. However, this approach is problematic because it complicates between-study comparisons and because the results are entirely dependent on the particular set of samples that were analyzed. We sought to standardize and advance the assignment of samples to CSTs.

**Results:**

We developed VALENCIA (*VA*gina*L* community state typ*E N*earest *C*entro*I*d cl*A*ssifier), a nearest centroid-based tool which classifies samples based on their similarity to a set of reference centroids. The references were defined using a comprehensive set of 13,160 taxonomic profiles from 1975 women in the USA. This large dataset allowed us to comprehensively identify, define, and characterize vaginal CSTs common to reproductive age women and expand upon the CSTs that had been defined in previous studies. We validated the broad applicability of VALENCIA for the classification of vaginal microbial communities by using it to classify three test datasets which included reproductive age eastern and southern African women, adolescent girls, and a racially/ethnically and geographically diverse sample of postmenopausal women. VALENCIA performed well on all three datasets despite the substantial variations in sequencing strategies and bioinformatics pipelines, indicating its broad application to vaginal microbiota. We further describe the relationships between community characteristics (vaginal pH, Nugent score) and participant demographics (race, age) and the CSTs defined by VALENCIA.

**Conclusion:**

VALENCIA provides a much-needed solution for the robust and reproducible assignment of vaginal community state types. This will allow unbiased analysis of both small and large vaginal microbiota datasets, comparisons between datasets and meta-analyses that combine multiple datasets.

Video abstract.

**Supplementary information:**

**Supplementary information** accompanies this paper at 10.1186/s40168-020-00934-6.

## Introduction

It is human nature to group objects and observations into categories based on their commonalities [1]. Doing so allows us to identify similarities and provides a unified framework for thought. This approach is particularly useful when the underlying set of objects or observations are multidimensional and difficult to grasp through intuition. When confronted with the diversity present in the microbial communities that inhabit the human body, microbiome scientists have often turned toward categorization [2]. These communities routinely include hundreds of species with a long tail of taxonomic diversity [3]. Variation in the community composition between specific body sites and individuals can be high, leading many to suggest that each person has their own “microbial fingerprint” [4]. Yet commonalities exist in the taxonomic compositions of these diverse communities and have enabled their categorization into types. This approach has been widely applied to human enteric [5, 6], vaginal [7, 8], skin [9], lung [10], and oral microbial communities [11] and has provided critical insights into the structure, function, and epidemiology of the human microbiome.

Hierarchical clustering (HC) is perhaps the most common approach used to categorize microbiota based on their composition and is performed on a matrix containing the distance between all pairwise combinations of samples. This approach is problematic for at least two reasons. First, the samples are typically clustered only within the study, complicating the interpretation of the findings in the context of other studies. This is especially problematic given the increasing volume of studies published on the human microbiome [12]. Second, because HC relies on pairwise distances between samples, the categories provided are entirely dependent on the particular set of samples included in the clustering. This means that assignments derived from HC can be unstable [13]. Removing a single sample from the dataset has a cascading effect on the assignment of the remaining samples. To overcome these limitations, we developed a nearest centroid classification algorithm to reproducibly place microbial communities into categories based on their composition and structure. This approach leverages a training dataset to define the centroid of each category and then places new data into categories based on the centroid to which they bear the highest similarity. It has been used previously to categorize proteins based on mass spectrometry data [14] and tumor subtypes based on gene expression patterns [15]. The resulting assignments are not dependent on within-study comparisons and therefore do not suffer from the same limitations as those provided by HC. Nearest centroid classification assignments are generally robust and can be compared across studies.

To demonstrate the utility of the nearest centroid classification, we implemented it for the assignment of vaginal microbial community profiles to community state types (CSTs). The concept of CSTs was introduced in 2011 by Ravel et al. to categorize vaginal microbial communities routinely observed among reproductive age women [7], and built upon prior methods to categorize these communities [16]. That study, and many subsequent studies [7, 17–21], have indicated that there are at least five vaginal CSTs, four of which are each dominated by different *Lactobacillus* spp. and another characterized by a more even community of facultative and obligate anaerobic bacteria (some studies have also distinguished subtypes within this CST [22]). Additional longitudinal studies have demonstrated that there can be a high degree of variation in community composition within a reproductive age woman over time [22–24], making it more appropriate to think of CSTs as a snapshot of the community at the time of sampling (i.e. state type) rather than a “type” which implies it is static over time. The dimensionality reduction provided by the CST approach has allowed epidemiologists to link variation in the vaginal microbiota with vaginal inflammation [25], STI occurrence [21, 26, 27], *Candida* detection [28], signs of vaginal atrophy [29], and increased risk of preterm birth [30].

To develop our nearest centroid classifier, we leveraged a large dataset of vaginal bacterial community profiles as defined by 16S rRNA gene amplicon sequencing (> 13,000 samples from > 1900 women). This dataset allowed us to comprehensively identify, define, and characterize vaginal CSTs common to North American reproductive age women using HC. In doing so, we recapitulated and expanded upon the CSTs that had been defined in previous studies. We then constructed reference centroids and applied the nearest centroid classification algorithm for the assignment of vaginal microbiota profiles to CSTs. We demonstrated the utility and robustness of the resulting tool, VALENCIA (*VA*gina*L* community state typ*E N*earest *C*entro*I*d cl*A*ssifier), using several publicly available test datasets that contained vaginal samples from adolescent girls [31], postmenopausal women (from the Study of Women’s Health Across the Nation (SWAN)), and eastern and southern African women [32]. These test datasets were also derived from the sequencing of different 16S rRNA variable regions and different bioinformatics pipelines. Finally, we examine relationships between vaginal CSTs, as defined by VALENCIA, and host (race, age) and community (pH, Nugent score) characteristics. We anticipate that VALENCIA will prove a critical tool for vaginal microbiota research and potential future clinical application by providing robust and reproducible assignments of samples to CSTs.

## Results

### Assemblage of the largest dataset of human vaginal microbiota profiles

We compiled a dataset of vaginal community compositions from 13,160 vaginal swab or lavage specimens that had been collected by our research group from three locations around the USA: Baltimore, MD; Birmingham, AL; and Atlanta, GA. The samples originated from 1975 North American women and included participants who self-identified as Black (*n* = 1343, 68%), White (*n* = 403, 20.4%), Hispanic (*n* = 110, 5.6%), Asian (*n* = 95, 4.8%), as well as 17 women who identified as a different race and 7 women who did not self-identify. Many of the women had participated in longitudinal studies (*n* = 916) and therefore contributed more than one sample to the compiled dataset; the median number of samples contributed per participant was three and ranged from one to seventy. All of the women included in this study were of reproductive age as defined by recent menstruation and were not pregnant at the time of sampling. The participant age range was 13–53 with a median participant age of 25. Eleven of the women were younger than 15 and two were older than 49. The composition of their vaginal microbiota was established by deep sequencing of the V3–V4 region of the 16S rRNA gene with an average of 54,898 reads per sample (range 1005–411,805).

### Construction of community state type reference centroids

**Fig. 1:**
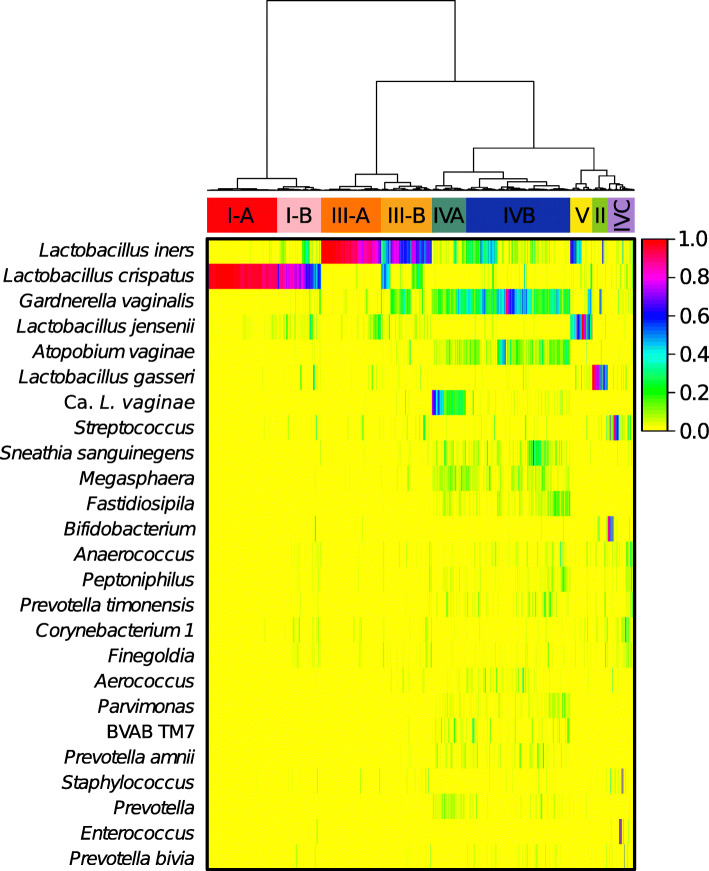
Heatmap displaying the taxonomic composition of 13,160 vaginal microbial communities using the 25 most abundant phylotypes across all samples. Hierarchical clustering was performed using Bray-Curtis dissimilarity with Ward linkage. Seven community state types were defined, four of which were dominated by a single species of *Lactobacillus* and three which were not. This dataset was used to train VALENCIA

The compiled dataset of 13,160 vaginal microbiota profiles includes representations of all previously identified CSTs and was used as a comprehensive training dataset. We first defined the CSTs in the training dataset using hierarchical clustering of the pairwise Bray-Curtis distances between samples with Ward linkage (Fig. 1). We then identified seven CSTs, four of which had a high relative abundance of *Lactobacillus* species. These seven CSTs could be further broken down into thirteen sub-CSTs. To conform with previous studies, we name these as follows: CST I*—L. crispatus* dominated, CST II—*L. gasseri* dominated, CST III—*L. iners* dominated, and CST V—*L. jensenii* dominated. CSTs I and III were more common in this dataset than CSTs II and V and were each split into two sub-CSTs denoted with A and B. The “A” version represents samples that had a higher relative abundance of the focal species, with the “B” version representing samples with a somewhat lower relative abundance of that species. We also identified three CSTs which did not have a high relative abundance of lactobacilli which we term CST IV-A, IV-B, and IV-C. CST IV-A had a high relative abundance of *Candidatus Lachnocurva vaginae* (formerly known as BVAB1 [33]) and a moderate relative abundance of *G. vaginalis*, while IV-B had a high relative abundance of *G. vaginalis* and low relative abundance of *Ca. L. vaginae*. Both IV-A and IV-B had moderate relative abundances of *Atopobium vaginae*. Samples assigned to CST IV-C had a low relative abundance of *Lactobacillus* spp., *G. vaginalis*, *A. vaginae*, and *Ca. L. vaginae* and were instead characterized by the abundance of a diverse array of facultative and strictly anaerobic bacteria. We thus further split CST IV-C into 5 sub-CSTs as follows: CST IV-C0—an even community with moderate amount of *Prevotella*, CST IV-C1—*Streptococcus* dominated, CST IV-C2—*Enterococcus* dominated, CST IV-C3—*Bifidobacterium* dominated, and CST IV-C4—*Staphylococcus* dominated. Samples assigned to CST IV-C represented 6% (*n* = 802) of the training dataset.

**Fig. 2:**
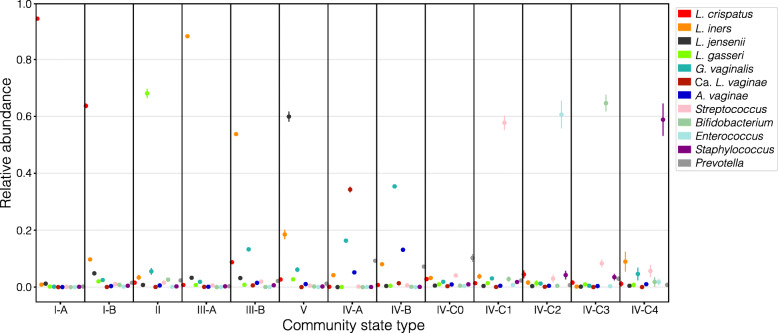
Average relative abundance of twelve key taxa across all of the samples used to define each of the thirteen sub-CSTs. Error bars represent the standard error of the mean as defined using 100 bootstraps of ten percent of the training dataset. These “average” communities define the reference centroids used by VALENCIA to assign new samples to sub-CSTs. Sub-CST IV-C0 is not dominated by any one species. CST V has 20% relative abundance of *L. iners* in addition to *L. jensenii*, indicating these two species can co-occur. This relationship is maintained over extended periods of time in some longitudinal profiles

We next constructed a reference centroid for each of the thirteen sub-CSTs identified in the training dataset by averaging the relative abundances of each taxa across the samples assigned to the sub-CST. These centroids represent the average community composition of each sub-CST, as defined by the training dataset, and can be used as a stable reference for the assignment of vaginal microbiota profiles to CSTs (Fig. 2).

### VALENCIA: a novel method for assigning samples to community state types

We implemented a nearest centroid classification algorithm, which we term VALENCIA, to leverage the training dataset for reproducible and robust assignment of vaginal microbiota to community state types. The similarity of a vaginal microbiota profile to each of the thirteen reference centroids is evaluated using Yue and Clayton’s *θ* [34], a similarity measure based on species proportions. This yields an array of thirteen similarity scores, ranging from 0.0 (no shared taxa) to 1.0 (all taxa shared and at the same relative abundance) for each sample. The reference centroid to which the sample bears the highest similarity provides an optimal assignment to a sub-CST. These similarity scores can also be used to gauge confidence in the assignment and are particularly useful for handling cases where the sample either does not match any of the centroids or has close matches to multiple centroids. For these cases, VALENCIA yields a low confidence score to indicate the degree of ambiguity in the CST assignment.

**Fig. 3:**
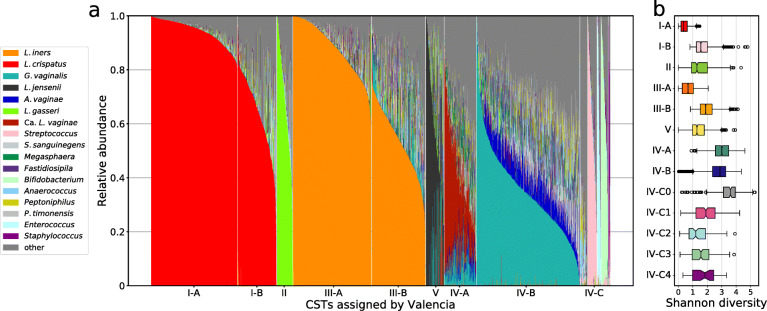
Taxonomic composition of all samples (*n* = 13,160) in the training data set categorized by sub-CST assignment according to VALENCIA (**a**). Distribution of Shannon diversity index values by sub-CST assignment (**b**). Shannon diversity was calculated using the log base 2

We first used VALENCIA to reassign CSTs to our training dataset. The taxonomic composition of samples assigned to each CST generally matched that of the associated reference centroid (Fig. 3a). As expected, differences in Shannon diversity (H) were observed among the sub-CSTs (Fig. 3b). Samples assigned to sub-CSTs defined by the dominance of a single phylotype were found to have lower values of H. A comparison between the initial hierarchal clustering and the assignments provided by VALENCIA revealed 1454 disagreements (11% of the samples). Discordant samples largely originated from communities which bore some degree of similarity to multiple CSTs. These communities exist in the grey areas between two or more community state types and are therefore difficult to classify. Based on the taxonomic profiles for these discordant samples, we have more confidence in the assignment provided by VALENCIA than that provided by hierarchical clustering (Additional file 1). For example, a number of samples were assigned to CST V by the hierarchical clustering but only contained ~ 20% relative abundance of *L. jensenii* and were instead majority *L. iners*. VALENCIA assigned these samples to CST III-B, which we find more agreeable. A similar pattern can be seen for discordant sample hierarchical clustering assigned to CST II which, based on the reference centroid for this community state type, should have a majority of *L. gasseri*. In this case, the discordant CST II samples had either a majority *L. iners*, which VALENCIA assigned to CST III-B or a majority *G. vaginalis*, which VALENCIA assigned to CST IV-B.

### Validation of VALENCIA against outside datasets

**Fig. 4:**
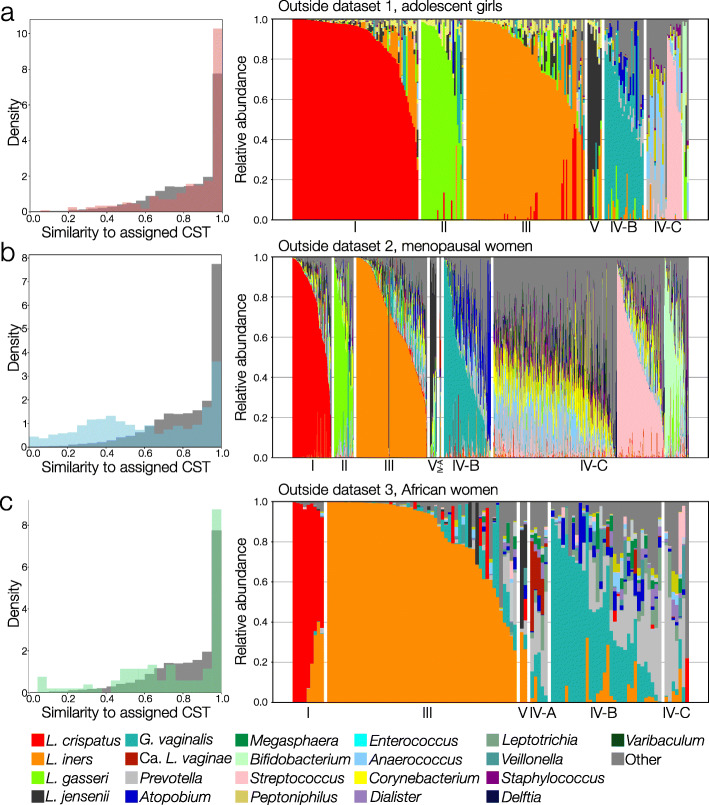
Validation of VALENCIA using three test datasets of vaginal taxonomic profiles derived from sequencing of the 16S rRNA gene. For each dataset, the similarity of each sample to its assigned sub-CST is plotted as a normalized histogram (left, **a** red, **b** blue, **c** green) versus that for the training dataset (dark grey). The taxonomic composition of each sample in the dataset is also provided (right). Test dataset 1 (**a**) was published by Hickey et al., contained 245 samples, was derived from sequencing of the V1V3 region, and contained samples from adolescent girls. Test dataset 2 (**b**) contained 1380 samples from menopausal women and was derived from sequencing of the V3V4 region. Test dataset 3 (**c**) was published by McClelland et al., contained 110 samples from eastern and southern African women, and was derived from sequencing of the V4 region

To further demonstrate the broad applicability of VALENCIA, we applied it to a number of test datasets that had not been included in the training dataset. These test datasets are from studies which had sampled women outside the age range of the training dataset, from non-North American populations, or had sequenced different 16S rRNA gene variable regions. Here we present our analysis of three such datasets: Test dataset 1 contained publicly available microbiota profiles from adolescent girls (*n* = 245, aged 10 to 15) derived from sequencing the 16S rRNA gene V1–V3 region [31]. Test dataset 2 was generated from SWAN analyzed *in-house* and contained microbiota profiles from postmenopausal women (*n* = 1380, aged 60 to 72) derived from sequencing the 16S rRNA gene V3–V4 region. Finally, test dataset 3 contained publicly available microbiota profiles from eastern and southern African reproductive age women (*n* = 110) derived from sequencing the 16S rRNA gene V4 region [32]. For test dataset 2, we applied our own taxonomic annotation pipeline to the data while for test datasets 1 and 3 we relied on the taxonomic annotations provided in the original studies. We assigned all of the samples in each of these three datasets to community state types using VALENCIA. In general, we find the CST assignments made by VALENCIA to be acceptable, although there is no “ground truth” from which to benchmark (Fig. 4). The distributions of similarity scores between the samples and their matching reference centroid for outside datasets 1 and 3 did not substantially differ from the same distribution provided by the reclassification of the training dataset (Fig. 4). Similarity scores for test dataset 2 were typically lower than those for the training dataset, although this is primarily driven by the high prevalence of CST IV-C and low prevalence of *Lactobacillus* spp.-dominant CSTs in postmenopausal women. For test dataset 1, the authors made their CST assignments (which were derived from hierarchical clustering) publicly available [31]. We find 17 instances of discordance between our and the original assignments (6.7%). As was the case for the reclassification of the training dataset, these discordant samples occupy the grey space between CSTs. For example, six instances of discordance come from communities which were assigned to CST I by Hickey et al. but had more *L. iners* than *L. crispatus*—VALENCIA assigned these samples to CST III-B.

### Relationships between VALENCIA-defined community state types, Nugent score, and vaginal pH

**Fig. 5:**
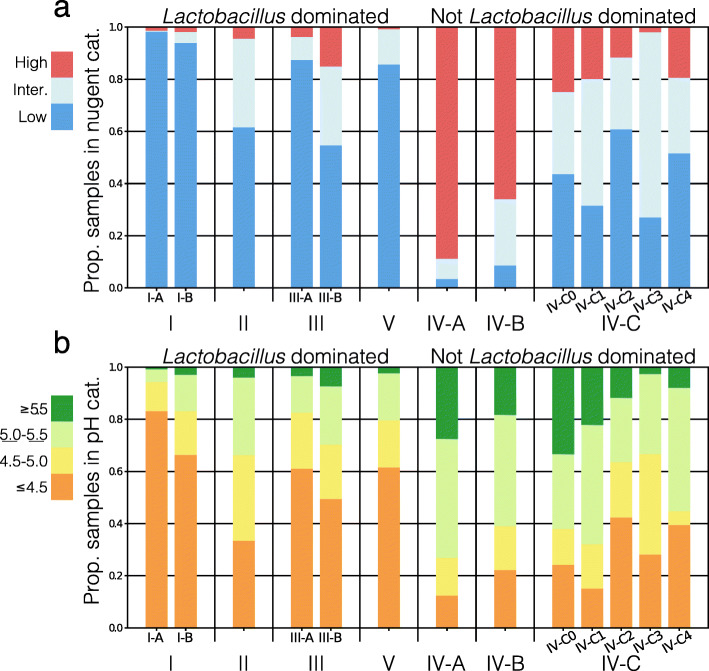
The relationship between each VALENCIA-assigned sub-CST and Nugent score (**a**) and vaginal pH (**b**). Nugent score was separated into high (score 8–10), intermediate (score 4–7), and low (score 0–3) categories. Vaginal pH was split into four categories: less than or equal to 4.5, between 4.5 and 5.0, between 5.0 and 5.5, and greater than or equal to 5.5

Prior to the application of amplicon sequencing to define microbiota composition, and still today, researchers and clinicians used the Nugent scoring system to evaluate and categorize vaginal microbial communities for the diagnosis of bacterial vaginosis (BV), a common vaginal condition [35]. The Nugent score is primarily based on the morphology of Gram-stained bacterial cells viewed under light microscopy. An abundance of *Lactobacillus* morphotypes, large Gram-positive rods, yields a low Nugent score while the presence of Gram-variable small (*G. vaginalis*) and/or curved rods (*Ca. L. vaginae* [36], *Mobiluncus* spp.) yields a high Nugent score, indicating Nugent-BV. We examined the relationship between VALENCIA-defined CSTs and Nugent score categories and observed high concordance (Fig. 5a). *Lactobacillus*-dominant CSTs typically have low Nugent scores, while communities assigned to other CSTs have higher Nugent scores. CST IV-A VALENCIA-assigned communities, which are enriched of *Ca. L. vaginae*, had the highest Nugent scores, consistent with the Nugent scoring system [37]. However, we were interested to see how the Nugent score system evaluated communities VALENCIA assigned to CST IV-C. These communities do not have a high relative abundance of *Lactobacillus* spp., *G. vaginalis*, or *Ca. L. vaginae* and instead have a high relative abundance of *Streptococcus*, *Enterococcus*, *Staphylococcus*, *Prevotella*, and *Bifidobacterium*. We find that the Nugent scoring system does not reliably assign these communities to a single category and instead gives mixed results. Of the five subtypes of CST IV-C, CST IV-C2 (*Enterococcus*), and IV-C4 (*Staphylococcus*) are most often assigned low Nugent scores although these communities are not dominated by *Lactobacillus* spp. Besides the ambiguities observed for these CST IV-C communities, VALENCIA-defined CSTs and the Nugent scoring system are largely in accordance with the definition of the Nugent score.

The microbiota is thought to be the primary driver of vaginal pH through the release of acidic fermentation end products. A low vaginal pH (≤ 4.5) has been associated with decreased risk of adverse health outcomes and is usually achieved via the production of lactic acid by lactobacilli [38–40]. Not surprisingly then, we found that the communities VALENCIA assigned to CSTs which are dominated by *Lactobacillus* spp. were associated with lower vaginal pH than those VALENCIA assigned to other CSTs (Fig. 5b, see supplemental figure 2 for odds of vaginal pH > 4.5 for each sub-CST). Communities which were dominated by *L. crispatus* (CST I) had the lowest pH (78% of samples had a pH ≤ 4.5) with those dominated by *L. iners* (CST III) and *L. jensenii* (CST V) following close behind. *L. gasseri*-dominated communities (CST II) were associated with the highest vaginal pH among the *Lactobacillus* spp.-dominant CSTs. Also as expected, communities which were deficient in *Lactobacillus* spp. typically had a higher vaginal pH. Communities VALENCIA assigned to CST IV-A had the highest pH followed closely by those assigned to CST IV-B. Due to the large number of samples included in this dataset, we were also able to examine pH for samples assigned to the less common CSTs. Among the subtypes of CST IV-C, we find that communities with *Enterococcus*, *Staphylococcus*, or *Bifidobacterium* were associated with lower vaginal pH while the majority of *Streptococcus* communities typically had a higher pH. None of these CST IV-C sub-CSTs (percent of samples with pH ≤ 4.5: IV-C0 24%; IV-C1 15%; IV-C2 42%; IV-C3 28%; IV-C4 39%) consistently reach the low vaginal pH achieved by CSTs I, III, or V (percent of samples with pH ≤ 4.5: I-A 83%; III-A 61%; V 61%, Fig. 5b).

### Associations between community state types and participant’s race and age

**Fig. 6:**
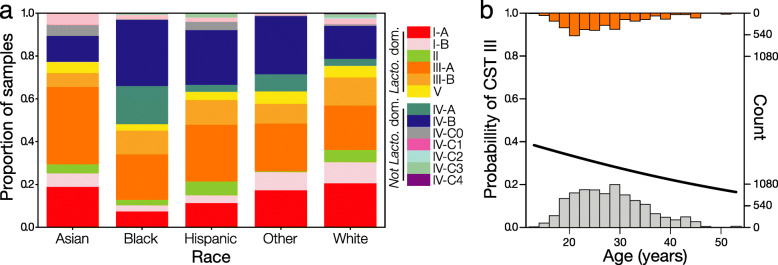
The relationship between the prevalence of each VALENCIA-assigned sub-CST and a woman’s self-identified race (**a**). Each bar represents the proportion of samples assigned to each CST in women whose race is Asian (*n* = 95), Black (*n* = 1,343), Hispanic (*n* = 110), White (*n* = 403), or Other (*n* = 17). For subjects who contributed multiple samples, the within subject relative prevalence of each CST was used in the calculation instead of their individual CST counts. We also examined relationships between the prevalence of each CST and a woman’s age (**b**). Only the prevalence of CST III was found to have a relationship with age among reproductive-age women. Bars represent the age distribution of subjects whose samples were (orange) or were not (grey) assigned to CST III. Older reproductive age women were less likely to have communities assigned to CST III than younger reproductive age women

Statistical associations between CSTs and demographic, medical, and/or behavior data have been used to identify factors that influence the composition of vaginal microbiota. Our training dataset contained microbiota compositions from over 1900 allowing us to re-examine some previously observed associations with more statistical power. We sought to determine whether a participant’s race or age influenced their likelihood of having particular community state types. For each community state type with sufficient sample size (I, II, III, IV-A, IV-B, IV-C), we modeled their presence or absence as a binomial response variable with race and age as fixed predictor variables and the subject as a random variable (Fig. 6a). We found that women who self-identify as Black or African American were less likely to have CST I than women who identify as White or Asian (*z* = 4.6, *p* < 0.001; *z* = 2.9, *p* = 0.0235). Black women were also more likely to have CST IV-A than White women (*z* = 4.9, *p* < 0.001) and CST IV-B than women who identified as White or Asian (odds ratios 6.91 versus 0.96 and 0.05; *p* < 0.001, *p* < 0.001). None of the Asian women included in this study were found to have CST IV-A. CST IV-B was also more common for Hispanic women than White women (*z* = 3.3, *p* = 0.006). Finally, we found that Asian women were more likely to have CST III than either Black or White women, although this association was weaker (odds ratios 1.72 versus 0.34 and 0.36; *p* = 0.025, *p* = 0.050). No significant associations with race were found for CSTs II, V, or IV-C, which may be due to sample size limitations as these three CSTs are less prevalent than the others. Our results agree with previous studies [7, 41] and show a more refined association between raise and the prevalence of CSTs.

We were also able to examine associations between a participant’s age and their likelihood of having each CST using the same logistic regression models. Our analyses indicate that the representation of CST III varies with age, after adjusting for race (Fig. 6b). Among reproductive age women, we observed that older women were less likely to have *L. iners-*dominated CSTs than younger women (*z* = − 3.589, *p* < 0.001). The probability of having CST III ranged from 40% for the youngest women included in the study, down to 20% for the oldest women. No significant associations with age were observed for the other CSTs. However, in our validation of VALENCIA, we analyzed the SWAN dataset of postmenopausal women between the ages of 60 and 72 (Fig. 4b). As expected, the representation of CSTs clearly differs between this dataset of postmenopausal women and the training dataset of reproductive age women.

## Discussion

The classification of vaginal microbial communities into CSTs has proven to be highly valuable. Since their inception in 2011 [7], studies have shown associations between vaginal CSTs and host immune profiles [25] and have further linked particular CSTs to sexually transmitted infections [18, 21, 26, 27] and experiencing spontaneous preterm birth [8, 42, 43]. From these studies, it is clear that the CST classification system captures meaningful information about these communities, despite its apparent simplicity. One of the advantages of the CST classification approach is that it enables the usage of standard and vetted statistical models to demonstrate these associations. On the other hand, a common criticism of the CST approach is that it simplifies the communities by distilling their composition down to a single categorical variable. One alternative has been to instead model the abundance of each taxon individually. However, these analyses are complicated by spurious correlations introduced because each taxa’s abundance is expressed relative to the others when assessed via amplicon sequencing [44]. Others have used species-specific quantitative PCR assays to determine their absolute abundances. Yet this method is expensive, requires the development and implementation of many individual qPCR assays, and only quantifies the targeted species. New statistical and methodological approaches are needed that can facilitate such taxa-level association studies which take into account the specific statistical challenges of compositional data [45]. Even then, there appears to be a place for “CST-level” analyses in vaginal microbiome research as the approach provides a higher-level overview and epidemiological characterization of the vaginal microbial community across populations and serves as a guide for more in-depth analyses.

In order for a classification scheme to be useful, it must be reproducible. It immediately becomes difficult to compare the results from multiple studies if the samples were not assigned to categories in the same manner. Prior work on assignment of vaginal microbial communities to CSTs has primarily been accomplished through within-study hierarchical clustering of the pairwise distances between samples, which does not yield reproducible assignments. Other studies have used taxa-specific relative abundance thresholds to assign CSTs (e.g., communities with > 30% relative abundance of *L. crispatus* are assigned to CST I [19, 46]). However, this approach does not consider the entire microbial community and may offer a limited view of the vaginal microbiota when only a few taxa are considered. VALENCIA provides a unified method to accomplish this task which is based on the overall structure and composition of the community. The CST assignments provided by VALENCIA are robust and reproducible across studies and will enable researchers to leverage the numerous existing vaginal microbiota datasets for use in large-scale meta-analyses. On the other end of the scale, we also expect that VALENCIA will assist the analysis of small datasets, which are often plagued by poor CST assignments. Because VALENCIA is reference-based, every sample is treated independently, making it scalable to large or small datasets—VALENCIA can even be used to assign a single sample to a CST. The structured nature of VALENCIA CSTs also allows the researcher to tune the number of CSTs considered to the size of the study (e.g., five, seven, nine or thirteen). Unlike previous classification methods, VALENCIA also provides an estimate of confidence derived from similarity of each sample to each reference centroid. These values can be used in a resampling scheme to investigate the effect of shuffling communities which bear similarity to multiple CSTs.

Overall, there is an astonishing concordance in the makeup of the vaginal microbiota. Most women were found to have communities which fit neatly within the CSTs that we have defined, driving the broad applicability of VALENCIA for the classification of vaginal microbiota. Despite this, we and others have shown that there are differences in the prevalence of particular CSTs that are associated with a woman’s race [7, 41]. Care should be taken in conveying precisely what these differences are and what they are not. In particular, we have found that women of African descent are less likely to have a *L. crispatus-*dominated community and more likely to have CST IV-B than women of European or Asian descent. It is important to note that these results are not consistent with there being distinct or systematic differences in the taxonomic composition of the communities. In this study, we identified every CST in women from each self-identified racial category with the exception of CST IV-A in Asian women. It is merely that some CSTs are more prevalent in women of a certain race while other CSTs are less prevalent. The factors that drive these differences in the representation of vaginal CSTs have yet to be determined, are likely to be multifaceted, and could depend on host and/or microbial factors. For example, our recent study indicated that there may be racial differences in the interplay between vaginal microbial communities and the host immune system [43].

The number of samples included in our training dataset allowed us to define and investigate some of the less prevalent types of *Lactobacillus*-deficient vaginal communities. We placed these communities into CST IV-C, and further defined five subtypes. Of these, CST IV-C1 (*Streptococcus*-dominated) and CST IV-C3 (*Bifidobacterium*-dominated) were the most common among reproductive age women. The Nugent scoring system was not designed with either of these taxa in mind [35] and it is not clear how they relate to vaginal health [47]. While group B *Streptococcus* is a known neonatal pathogen, it is not known whether its pathogenic potential is at all realized in the vaginal environment [48]. Like the lactobacilli, many *Streptococcus* spp. can produce lactic acid as a fermentation end product [49] and therefore might be able to lower vaginal pH to a similar degree. Alas, we found these communities to instead be associated with high vaginal pH, indicating that vaginal *Streptococcus* spp. might produce other fermentation end products in the vagina. *Bifidobacterium*, on the other hand, has a reputation for being a “healthy” microbe based on its activity in the gut environment [50]. Due to its rarity, associations between *Bifidobacterium*-dominated communities and vaginal health have yet to be assessed. Although they are generally capable of producing L-lactic acid [51], we found that only about a quarter of women with *Bifidobacterium*-dominated communities had a vaginal pH ≤ 4.5, indicating it likely does not provide the same level of pH-mediated protection as a *Lactobacillus*-dominated community. Going forward, we hope that VALENCIA will enable association studies between these two uncommon CSTs and vaginal health. However, both the *Streptococcus* and *Bifidobacterium* genera include a diverse array of described species. Species- and strain-level characterization using metagenomic approaches may be necessary to understand their ecology in the vaginal environment.

As other reference-based approaches, one potential limitation of VALENCIA is that it is not able to classify communities which were not included in the training dataset. Though VALENCIA was demonstrated to be applicable on different populations, age ranges, and 16S rRNA regions, misclassification could happen for samples with novel bacteria or different community structures. The only potential issues we observed were related to the presence of community profiles which did not completely match those in the reference. For example, CST IV communities from some African populations tend to have a higher relative abundance of *Prevotella* spp. than CST IV communities from North American women [21, 25, 32]. We have shown, in our analysis of test dataset 3, that VALENCIA assigns these communities to one of the subtypes of CST IV depending on the presence and abundance of other taxa. However, an argument could be made for the addition of a novel CST and reference centroid which is *Prevotella*-dominated as defined by samples from African women. VALENCIA can be expanded by the addition of novel CSTs not found in the training dataset, when appropriate. This could be achieved either by adding data defining these novel CSTs to the existing training dataset or by constructing new training datasets from samples originating from a specific population of interest. As we collect more data from women across the globe, we plan to expand, update, and maintain VALENCIA.

We used the nearest centroid categorization approach to assign vaginal microbiota profiles to community state types. Our training and test datasets were all derived from 16S rRNA gene amplicon sequencing, but VALENCIA could also be used to categorize vaginal communities based on composition as established by shotgun metagenome data. This would likely not require additional changes to the tool or training dataset. In addition to this natural extension, a similar nearest centroid approach could be applied to the classification of other microbial communities into types. In many ways, vaginal microbiota are perhaps the easiest to categorize based on taxonomic composition because of their tendency to be dominated by a single species, which results in fairly distinct lines between community state types. The microbial communities which inhabit other body sites [52] or other environments (e.g., soil [53], ocean water [54]) tend to have communities which are more even and species rich. This is likely to blur the lines between community types and may complicate the use of the nearest centroid approach for their categorization. Microbial communities could also be categorized based on their functional and metabolomic composition. Classification of these multidimensional microbiome datasets would likely make their analysis and interpretation less complicated. A reference-based approach like the nearest centroid classification used here would provide robust and reproducible assignments.

## Conclusion

We used a large dataset of over 13,160 vaginal microbiota profiles to train a nearest centroid classifier (VALENCIA) to infer community state types. The large training dataset allowed us to define CSTs which represent more uncommon vaginal microbiota compositions (e.g., those dominated by *Bifidobacterium* spp.). Our validation efforts demonstrated that VALENCIA provides robust and reproducible assignments of vaginal microbiota profiles to CSTs that are insensitive to a women’s age or geographic location. VALENCIA assignments are also largely unaffected by which variable region of the 16S rRNA gene was sequenced or which bioinformatics pipeline was used to taxonomically identify the resulting sequences. We expect that VALENCIA will enable epidemiological investigations into the factors that drive changes in the vaginal microbiota and associations between these communities and vaginal health. The reproducibility of this approach will allow for much-needed meta-analyses that combine the results of the myriad of existing studies on the vaginal microbiota.

## Methods

### Participants and sampling procedures

The training dataset of 13,160 vaginal microbiota profiles originated from several different studies, all of which have been published previously [7, 22, 55–59]. A detailed explanation of the sample procedures and study populations can be found in the original publications. Samples were either self-collected or physician-collected by swabbing the mid-vagina (*n* = 11,387) or physician collected via a vaginal lavage with 3 mL of sterile deionized water (*n* = 1844). Vaginal swabs and vaginal lavage samples were frozen at − 80 °C. Participants also provided behavior and lifestyle information. Nugent scoring was performed as previously described [7, 35]. Vaginal pH was established using the ﻿VpH glove (Inverness Medical) and binned into categories (≤ 4.5, greater than 4.5 but less than 5.0, between 5.0 and 5.5 inclusive, and ≥ 5.5). All studies were performed under Institutional Review Board-approved protocols, and samples were collected after obtaining written informed consent from all the participants.

### DNA extraction

DNA was extracted from the samples using a combination of enzymatic digestion and mechanical disruption as described in Holm et al. [60]. Briefly, vaginal swab or lavage specimens were resuspended in phosphate buffer saline solution. A 0.5 mL aliquot of the cell suspension was incubated at 37 °C for 30 min following the addition of an enzymatic digestion cocktail (contents: 5 μl of lysozyme at 10 mg/ml, EMD Chemicals, Gibbstown, NJ; 13 μl of mutanolysin at 11,700 U/ml; Sigma-Aldrich, St. Louis, MO; and 3.2 μl of lysostaphin a 1 mg/ml; Ambi Products, LLC, Lawrence, NY). This was followed by the addition of 10 μl of Proteinase K (20 mg/ml; Invitrogen), 50 μl of 10% SDS (Sigma-Aldrich, St. Louis, MO), and 2 μl of RNase A (10 mg/ml; Invitrogen, Carlsbad, CA) and a further 45 min of incubation at 55 °C. Mechanical disruption was then performed using a FastPrep homogenizer at 6 m/s for 40 s. DNA was then purified from the crude lysates using the QS DSP virus/pathogen midi kit on the QIAsymphony robotic platform (Qiagen, Hilden, Germany) according to the manufacturer’s specifications.

### 16S rRNA gene amplification, sequencing, and analysis

PCR amplification of the V3V4 region of the 16S rRNA gene was conducted using either the “one-step” or the “two-step” amplification protocols described and validated in Holm et al. [60]. Primer sequences can be found in supplemental table 1. For the one-step protocol, extracted DNA (0.5 μl) was added to Phusion Taq master mix (ThermoFisher, Waltham) with 3% dimethylsulfoxide (DMSO) and each primer (final concentration 0.4 μM). Initial denaturation was performed at 98 °C for 30 s, followed by 30 cycles of denaturation (98 °C, 15 s), annealing (58 °C, 15 s), and extension (72 °C, 15 s). Final extension was conducted at 72 °C for 60 s. The two-step protocol utilized two rounds of PCR amplification: the first amplifies the V3V4 region of the 16S rRNA gene and adds Illumina sequencing primers, and the second adds 8-bp barcode sequences. Both two-step reactions utilized the same Phusion Taq master mix with 3% DMSO and primers at 0.4 μM. The first round also included 0.5 μl of extracted DNA. Initial denaturization was conducted at 94 °C for 3 min, followed by 20 cycles of denaturation (94 °C, 30 s), annealing (58 °C, 30 s), extension (72 °C, 1 min), and a final extension step at 72 °C for 7 min. The second round of amplification used 1 μl of 1:20 diluted PCR product from the first round as input. Initial denaturation was conducted at 94 °C for 30 s, followed by 10 cycles of denaturation (94 °C, 30 s), annealing (58 °C,30 s), extension (72 °C, 60 s), and then a final extension (72 °C, 5 min). Deep sequencing was accomplished on either an Illumina MiSeq or an Illumina HiSeq 2500 [60]. The resulting paired end sequences were processed using DADA2 [61] to identify amplicon sequence variants (ASVs) and remove chimeric sequences following general practices (﻿https://benjjneb.github.io/dada2/bigdata.html). Following processing by DADA2, each sample had an average of 54,898 reads (range 1005–411,805). Taxonomy was assigned to each ASV using the RDP Naïve Bayesian Classifier [62] trained with the SILVA 16S rRNA gene database [63]. For several key genera (e.g., *Lactobacillus*, *Prevotella*, *Sneathia*, *Mobiluncus*), the ASVs were further classified to the species level using speciateIT (version 1.0, http://ravel-lab.org/speciateIT). Read counts for ASVs that were assigned to the same phylotype were combined. The final dataset contained 199 taxa following removal of those we identified as contaminants as well as those taxa present at a frequency of less than 10^−5^ study wide. The same bioinformatics procedures were used to analyze the *in-house* test dataset 2.

### Construction of the reference centroids

Hierarchical clustering of the 13,160 taxonomic profiles using Bray-Curtis distances and ward linkage was first employed to define the vaginal CSTs (Fig. 1). This analysis recovered the canonical five CSTs as described in Ravel et al. [7] but went further in delineating subtypes among the five CSTs. Cluster selection was made using the cutree function from the R stats package (version 3.6.0) on the dendrogram produced by hierarchical clustering. Cluster numbers from 2 to 20 were produced and then evaluated using the Davies Bouldin score. Support was found for nine clusters (Supplemental Figure 3c). For the *L. crispatus*-dominated CSTs, we were able to distinguish between communities which had mostly just *L. crispatus* (CST I-A) and those that had a lower, moderate relative abundance of the species (CST I-B). The same paradigm was observed for *L. iners-*dominated communities. Communities dominated by *L. gasseri* and *L. jensenii* more uncommon and were therefore not split into sub-CSTs. We were also able to distinguish three non-CSTs with a paucity of lactobacilli: CST IV-A, which contained *Ca. L. vaginae*, *G. vaginalis*, *A. vaginae*, and *Prevotella*; CST IV-B which contained *G. vaginalis*, *A. vaginae*, and *Prevotella*; and CST IV-C which was characterized by a paucity of *Lactobacillus* spp., *G. vaginalis*, *Ca. L. vaginae*, and *A. vaginae*. A second round of hierarchical clustering was performed (Bray-Curtis distances, ward linkage) on just the CST IV-C communities to further split this diverse collection of communities into additional sub-CSTs. Cutree was used on the resulting dendrogram to split IV-C into five subtypes, four of which had a characteristic phylotype and one which had a more even taxonomic composition. This decision improved assignments to IV-C, enhanced the interpretation of these communities, and resulted in a better Davies Bouldin score (Supplemental Figure 3). Reference centroids were constructed by averaging the relative abundances of each phylotype across the samples in training dataset which were included in each of the 13 sub-CSTs. Shannon diversity of samples assigned to each sub-CST was calculated using the log base 2.

### Implementation of the nearest centroid classification

VALENCIA uses the nearest centroid approach to classification to assign new samples to sub-CSTs based on their taxonomic composition and was implemented in python (version 3.6) and has the *pandas* module as a dependency [64]. The similarity of each sample to each reference centroid is assessed using Yue-Clayton’s *θ* [34], which considers the number and proportion of shared and unique phylotypes in its measure of similarity. Compared to Bray-Curtis or Jensen-Shannon, the Yue-Clayton *θ* measure depends more on the high relative abundance phylotypes than those that are at lower relative abundances. Samples are assigned to the sub-CST to which they bear the highest similarity and the degree of similarity to that sub-CST can be taken as a measure of confidence in the assignment. VALENCIA reports which sub-CST a sample was assigned to, as well as the set of similarity scores to each of the thirteen sub-CSTs. Also included in the output is a higher-order CST assignment which does not differentiate between the subtypes of I, III, or IV.

### Running VALENCIA

The expected input of VALENCIA is a table of taxa read counts in each sample with the phylotypes as columns and the samples as rows. The first column should contain a unique identifier for the sample with the column heading “sampleID”. The second column should contain the total read count for the sample with the column name “read_count”. The remaining columns should contain the read count for each phylotype in the dataset. It is *imperative* that phylotype column headings match those used by the VALENCIA reference centroids which generally take the form of “phylotype rank underscore phylotype name” (e.g., g_Bifidobacterium). All phylotypes should be summarized to the genus rank or higher except for the following: *Lactobacillus* spp., *Gardnerella* spp., *Prevotella* spp., *Atopobium* spp., *Sneathia* spp, *Mobiluncus* spp. These key phylotypes appear as “Genus underscore species” (e.g., Lactobacillus_crispatus, Gardnerella_vaginalis). The other required input is a provided file which contains the reference centroids. The expected output is a modified version of the input data table with added columns indicating the CST and sub-CST designations, the similarity of the sample to the assigned CST, and the array of similarities scores to all of the reference centroids. The tool also generates a figure illustrating the performance of VALENCIA on the dataset which displays the distribution of similarity scores for samples assigned to each sub-CST, compared to the median similarity score for the sub-CST in the training dataset. Substantial differences may indicate either an incongruence in taxonomic names or the need for an additional sub-CST and associated centroid. VALENCIA, the reference centroids, and the training dataset are all available at github.com/ravel-lab/VALENCIA.

### Validation efforts

Although there is no “gold-standard” to benchmark VALENCIA against, we did perform a number of tests to validate its use for the classification of vaginal microbial communities. First, we reclassified the training dataset using VALENCIA and compared the new assignments to those provided by the initial HC. We also tested the use of VALENCIA on other populations and on taxonomic compositions that had been generated by the sequencing of other 16S rRNA variable regions and other bioinformatics pipelines. Three datasets were used—two were published and made available by other groups and one which had been generated *in-house*. Test dataset 1 was published by Hickey et al in 2015 [31] and contained samples from adolescent girls aged 12–15. Test dataset 2 was generated in-house and contained samples from menopausal women above the age of 60. These data are available at github.com/ravel-lab/VALENCIA. Test dataset 3 was published by McClelland et al. [32] and contained samples from reproductive age eastern and southern African women. Test dataset 1 was derived from sequencing the V1V3 region of the 16S rRNA gene, test dataset 2 from the V3V4 region, and test dataset 3 from the V4 region. For test datasets 1 and 3, the published taxonomic assignments were used with adjustments to match the phylotype naming scheme used by VALENCIA. The *in-house* data included in test dataset 2 was generated via the same methods used in the generation of the training dataset. All three test datasets were classified using VALENCIA, the results of which are shown in Fig. 4. Although many factors can introduce bias in the assessment of taxonomic composition (e.g., DNA extraction, PCR primer selection [65, 66]), we did not find substantial irregularities in the assignment of CSTs by VALENCIA on these test datasets.

### Statistical analysis

Associations between the representation of each CST (I, II, III, IV-A, IV-B, IV-C, V) and a participant’s race and age were tested using separate generalized logistic regression models. The presence or absence of each CST was used as a response variable and the participant’s race and age were included as categorical and continuous predictor variables, respectively. Because many of the participants had included multiple samples, participant was also included as a random effect. The glmer function from the lme4 package [67] (version 1.1-21) for R [68] (version 3.6.0) was used with the bobyqa optimization function and 10^5^ iterations. Effect sizes were exponentiated using the R package broom.mixed [69] (version 0.2.4). All scripts used in the statistical analysis are available at github.com/ravel-lab/VALENCIA. The same approach was used to model the association between vaginal pH and each sub-CST. A single generalized logistic regression model was used to model vaginal pH as a binary variable with categories: ≤ 4.5 or > 4.5. The VALENCIA-defined sub-CST was used as a fixed predictor variable along with the subject as a random effect.

## Supplementary information

**Additional file 1: Supplemental Figure 1.** Illustration of cluster selection for construction of VALENCIA sub-CST centroids. Stretched version of dendrogram from Figure 1 with horizontal line indicating chosen cluster threshold (a). Subset heatmap and dendrogram of samples in CST IV-C (b). Hierarchical clustering was performed on this subset using Bray-Curtis dissimilarity with Ward linkage and the horizontal line indicating cluster threshold. Silhouette and Davies-Bouldin scores for a range of number of clusters (c). Horizontal lines indicate the values for the final 13 sub-CSTs, after sub-clustering the CST IV-C samples.

**Additional file 2: Supplemental Figure 2.** Odds of women with each sub-CST having a vaginal pH >4.5.

**Additional file 3.** Supplemental Figure 3.

**Additional file 4.** Table S1

## Data Availability

VALENCIA, the training dataset, test dataset 2, and all scripts used to analyze and display the data are available at https://github.com/ravel-lab/VALENCIA. Information on how to obtain test datasets 1 and 3 can be found in their respective papers.

## Abbreviations

CST: Community state type
HC: Hierarchical clustering
