# Supplementary material for: VALENCIA: a nearest centroid classification method for vaginal microbial communities based on composition
Source: Microbiome. 2020 Nov 23;8:166. doi: 10.1186/s40168-020-00934-6 (PMC7684964; doi:10.1186/s40168-020-00934-6)
Supplement: Supplementary file 2 — Additional file 1: Supplemental Figure 1. Illustration of cluster selection for construction of VALENCIA sub-CST centroids. Stretched version of dendrogram from Figure 1 with horizontal line indicating chosen cluster threshold (a). Subset heatmap and dendrogram of samples in CST IV-C (b). Hierarchical clustering was performed on this subset using Bray-Curtis dissimilarity with Ward linkage and the horizontal line indicating cluster threshold. Silhouette and Davies-Bouldin scores for a range of number of clusters (c). Horizontal lines indicate the values for the final 13 sub-CSTs, after sub-clustering the CST IV-C samples. [file 40168_2020_934_MOESM1_ESM.docx]

Additional File 1: Stacked bar plots displaying the taxonomic profiles of samples which were discordantly assigned community state types (CSTs) by hierarchical clustering (HC) and VALENCIA. Each plot contains samples assigned to the CST identified in the title and their VALENCIA assignments appear along the x-axis.
