# Supplementary figures and images for: VALENCIA: a nearest centroid classification method for vaginal microbial communities based on composition
Source: Microbiome. 2020 Nov 23;8:166. doi: 10.1186/s40168-020-00934-6 (PMC7684964; doi:10.1186/s40168-020-00934-6)

a

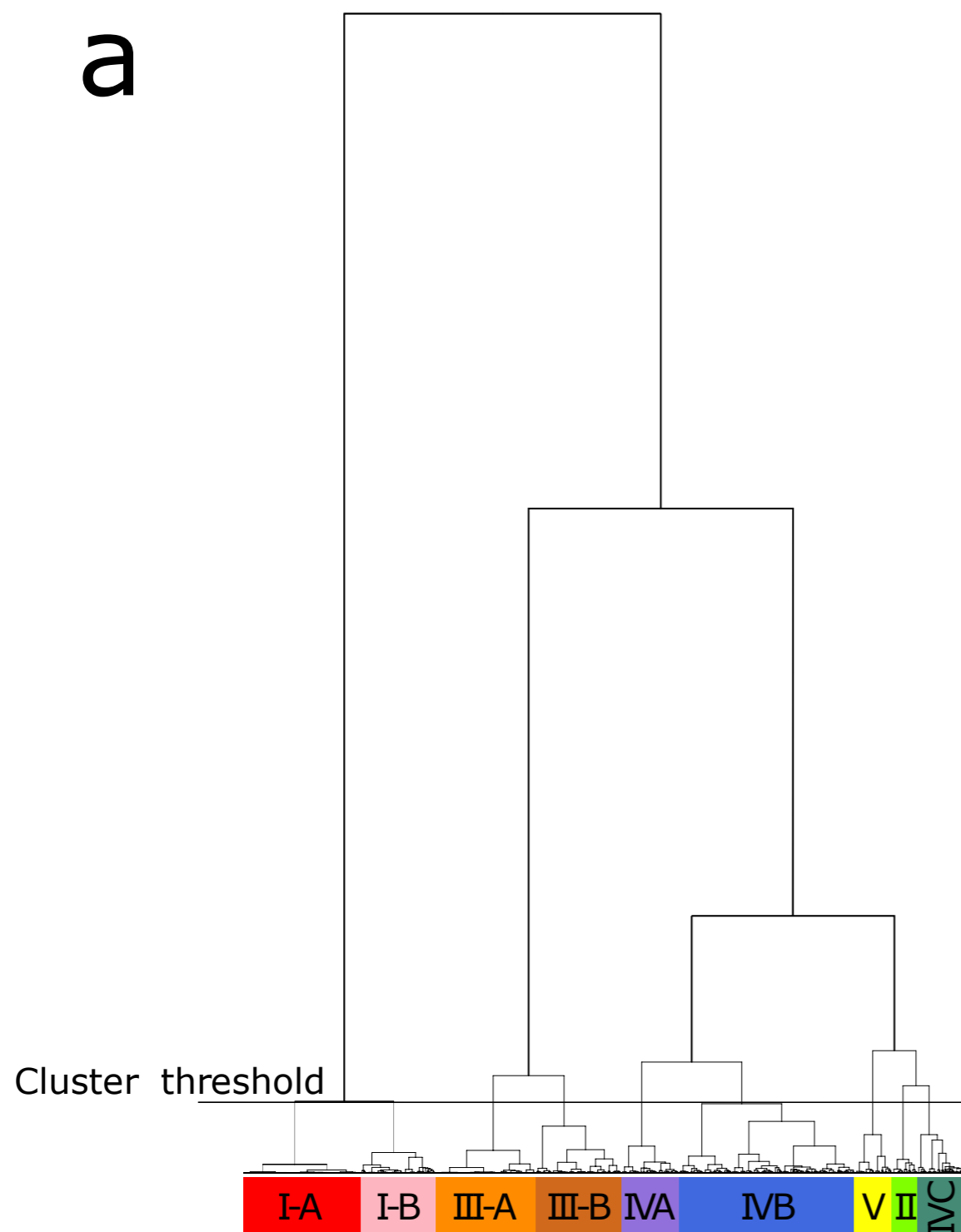

b

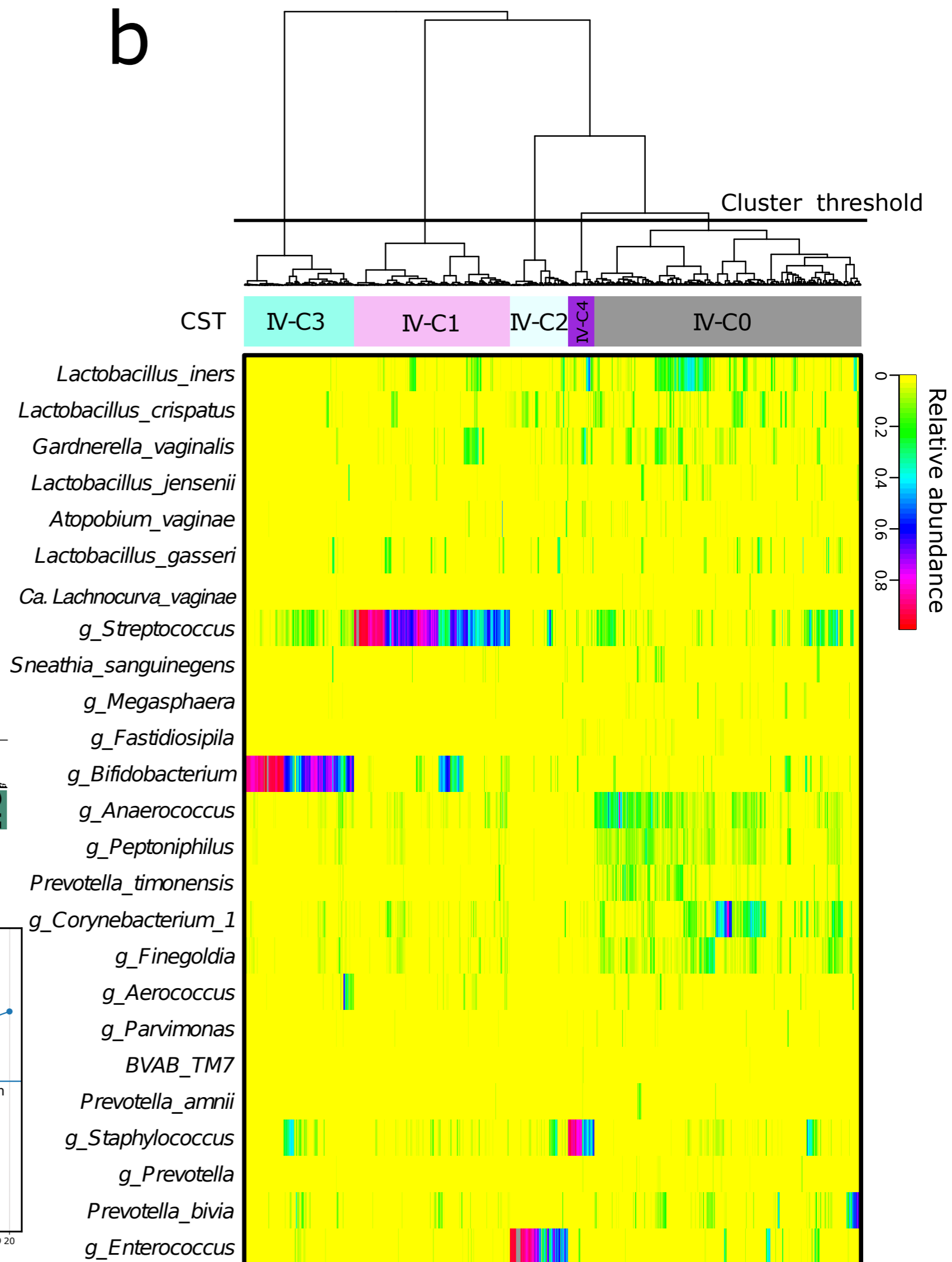

c

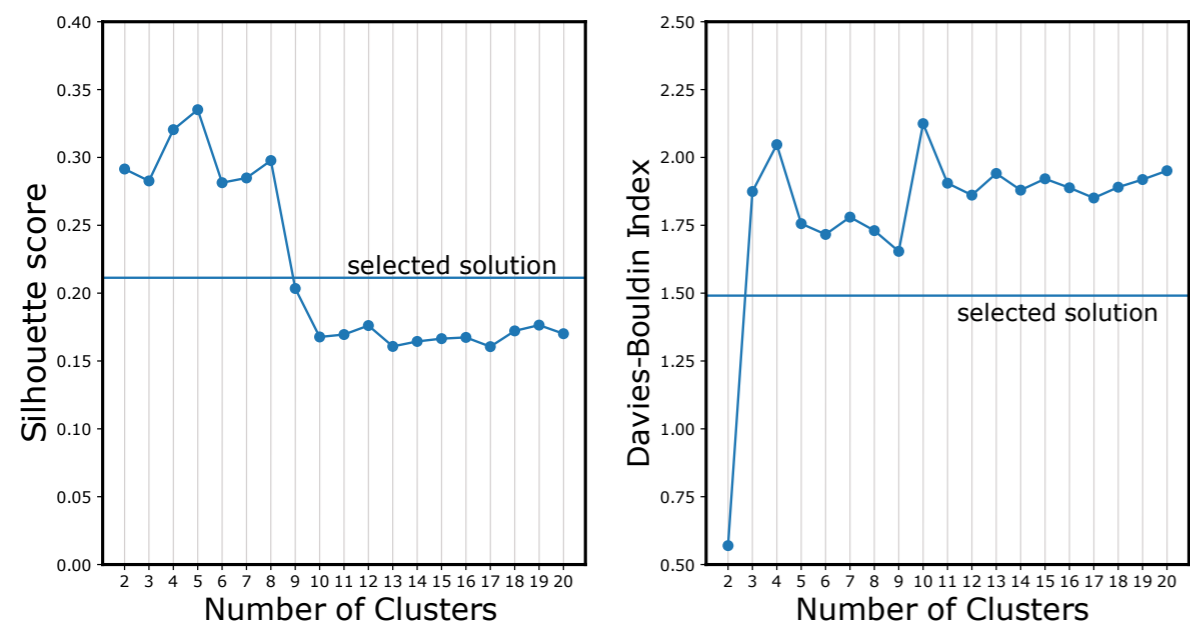

Supplement: Supplementary file 3 — Additional file 2: Supplemental Figure 2. Odds of women with each sub-CST having a vaginal pH >4.5. [file 40168_2020_934_MOESM2_ESM.pdf]

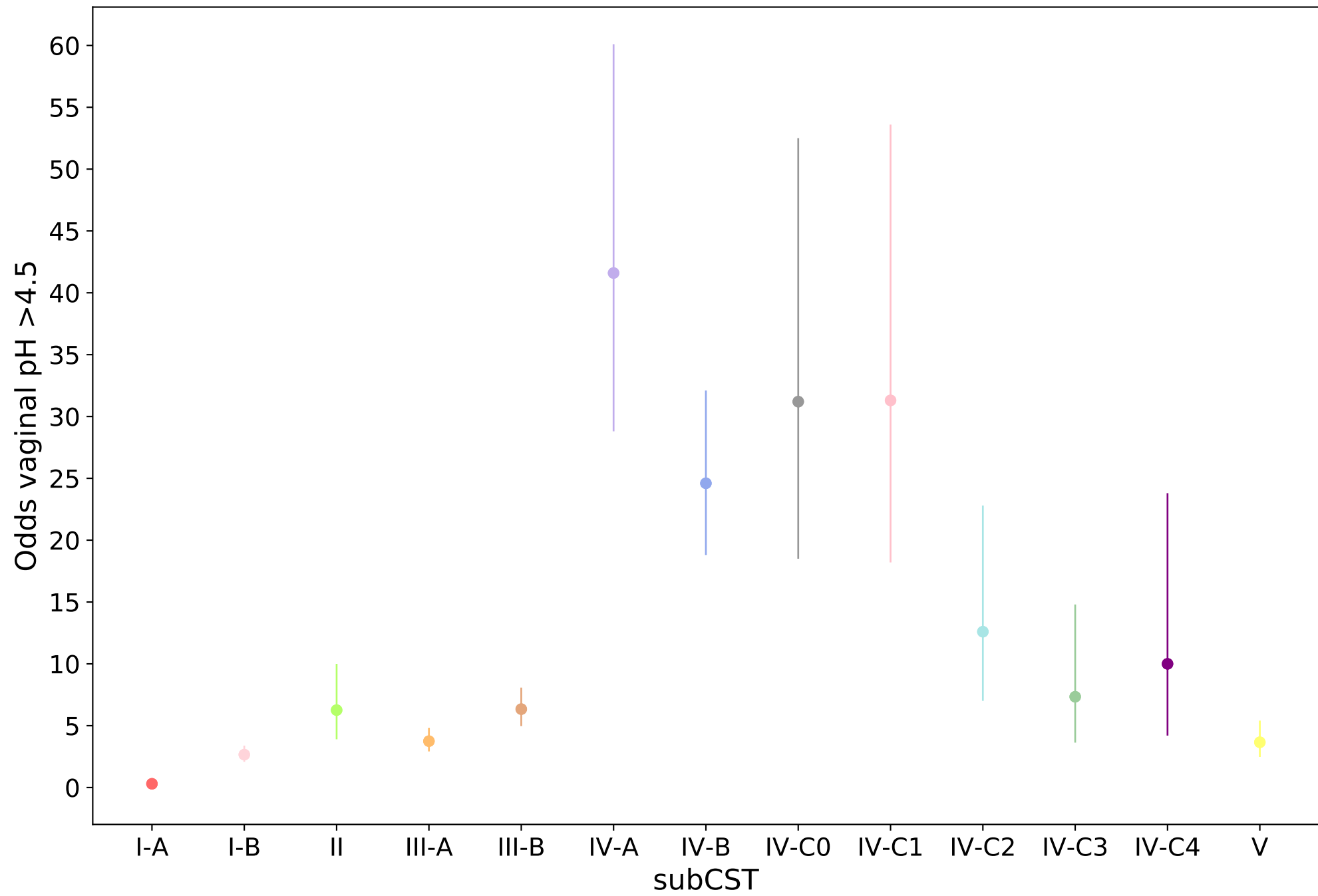

Supplement: Supplementary file 4 — Additional file 3. Supplemental Figure 3. [file 40168_2020_934_MOESM3_ESM.pdf]
